# Insulin-Producing Cells Derived from Human Embryonic Stem Cells: Comparison of Definitive Endoderm- and Nestin-Positive Progenitor-Based Differentiation Strategies

**DOI:** 10.1371/journal.pone.0072513

**Published:** 2013-08-12

**Authors:** Rui Wei, Jin Yang, Wenfang Hou, Guoqiang Liu, Meijuan Gao, Lin Zhang, Haining Wang, Genhong Mao, Hongwei Gao, Guian Chen, Tianpei Hong

**Affiliations:** 1 Department of Endocrinology and Metabolism, Peking University Third Hospital, Haidian District, Beijing, China; 2 Reproductive Medical Center, Peking University Third Hospital, Haidian District, Beijing, China; 3 Clinical Stem Cell Research Center, Peking University Third Hospital, Haidian District, Beijing, China; Children's Hospital Boston, United States of America

## Abstract

Human embryonic stem cells (hESCs) are pluripotent and capable of undergoing multilineage differentiation into highly specialized cells including pancreatic islet cells. Thus, they represent a novel alternative source for targeted therapies and regenerative medicine for diabetes. Significant progress has been made in differentiating hESCs toward pancreatic lineages. One approach is based on the similarities of pancreatic β cell and neuroepithelial development. Nestin-positive cells are selected as pancreatic β cell precursors and further differentiated to secrete insulin. The other approach is based on our knowledge of developmental biology in which the differentiation protocol sequentially reproduces the individual steps that are known in normal β cell ontogenesis during fetal pancreatic development. In the present study, the hESC cell line PKU1.1 was induced to differentiate into insulin-producing cells (IPCs) using both protocols. The differentiation process was dynamically investigated and the similarities and differences between both strategies were explored. Our results show that IPCs can be successfully induced with both differentiation strategies. The resulting IPCs from both protocols shared many similar features with pancreatic islet cells, but not mature, functional β cells. However, these differently-derived IPC cell types displayed specific morphologies and different expression levels of pancreatic islet development-related markers. These data not only broaden our outlook on hESC differentiation into IPCs, but also extend the full potential of these processes for regenerative medicine in diabetes.

## Introduction

Islet transplantation is a promising method to restore functional islet β cell mass for patients with diabetes [1]. Because of the limited supply of human donor islets, it is critical that new strategies are explored as alternative renewable sources of transplantation. Stem cells are characterized by extensive proliferation and multilineage differentiation capacity [2]. They may be a valuable source for cell replacement therapy. Human embryonic stem cells (hESCs) are capable of spontaneous differentiation into insulin producing cells (IPCs) [3]. In addition, significant progress has been made recently in inducing ESCs to preferentially differentiate into pancreatic lineages by changing the composition of the culture medium [4–8] and expressing dominant transcription factors involved in pancreas development [4,9–11].

To date, there are two main strategies for IPC differentiation of ESCs without genetic manipulation. One is based on the selection of nestin-positive progenitors [4,5], and the other is via the definitive endoderm (DE) route [6–8].

Pancreatic β cell specification depends on a succession of transcription factors that function in a marvelously coordinated, temporal, and spatial manner during pancreas development [12]. During *in vitro* differentiation of hESCs, this process may be mimicked through a multistep protocol by adding growth factors and/or chemical compounds that induce the proper expression of transcription factors at the opportune moment. Several recent studies have been successful in attempting *in vitro* differentiation of cells from pancreatic lineage. Reports by D’Amour et al. [8] and Jiang et al. [6] represent the most successful attempts. Based on our knowledge of basic developmental biology, the DE-based differentiation protocol sequentially reproduces the individual steps that characterize normal β cell ontogenesis [8].

Embryogenesis studies have shown that pancreatic cells do not originate from one source [13]. This suggests that other pathways lead to IPC production. Pancreatic β cell and neuroepithelial development is similar [14,15], and pancreatic β cells of endodermal origin share many common features with ectoderm-derived neurons, including transcription factors and biosynthetic enzymes, as well as secretory and metabolic proteins [16]. As such, transient expression of nestin has been proposed to occur in pancreatic precursors as seen in neuroepithelial differentiation [17]. In addition, several reports have demonstrated that differentiation of ESCs into IPCs can be successfully induced by selecting nestin-positive cells [4,5,9,18].

Both DE- and nestin-positive progenitor-based protocols are efficacious in inducing hESC differentiation into IPCs. However, it is still debated which approach is better suited for the treatment of diabetes. Until now, there are no data comparing the two protocols within the same laboratory. Moreover, the hESC cell lines exhibit a marked propensity to differentiate into the specific lineages [19]. Therefore, it is highly necessary to analyze the differences of these two protocols in the same hESC cell line for pancreatic β cell differentiation.

In the present study, we compared the DE and nestin protocols by documenting the similarities and differences between the two differentiation processes. We confirm that IPCs can be successfully induced using either strategy. The IPCs derived from both protocols had characteristic human pancreatic islet cell function, but not mature β cell function. Furthermore, these two different protocol-derived IPCs showed specific morphologies and different expression levels of pancreatic islet development-related markers. These data extend our understanding of the differentiation of hESCs into IPCs and suggest that further studies should focus on the differentiation reliability and maturity of IPCs.

## Materials and Methods

### Ethics Statement

The hESC cell line PKU1.1 was established at the Reproductive Medical Center of Peking University Third Hospital [20]. The mouse β cell line Min6 was provided by Prof. Yiming Mu (Chinese PLA General Hospital), and is derived from pancreatic β cell tumors [21]. The human adult islet RNA was a gift from Prof. Jinning Lou (China-Japan Friendship Hospital) [22,23].

### hESC culture and differentiation

The hESC cell line PKU1.1 exhibits a normal female karyotype (46, XX) [20]. The cells were cultured on γ-ray irradiated mouse embryonic fibroblast feeder layers in hESC medium under 5% CO_2_ in air at 37° C. The hESC medium contained KnockOut^TM^ Dulbecco’s modified Eagle’s medium (Invitrogen) supplemented with 20% (v/v) KnockOut^TM^ serum replacement (SR, Invitrogen), 1% (v/v) nonessential amino acids (NEAA, Invitrogen), 2 mM GlutaMAX (Invitrogen), 4 ng/ml basic fibroblast growth factor (bFGF, Peprotech) and 0.1 mM β-mercaptoethanol (Invitrogen). The cell colonies were passaged every 5-7 days by incubation in 1 mg/ml collagenase IV (Invitrogen).

**Figure 1:**
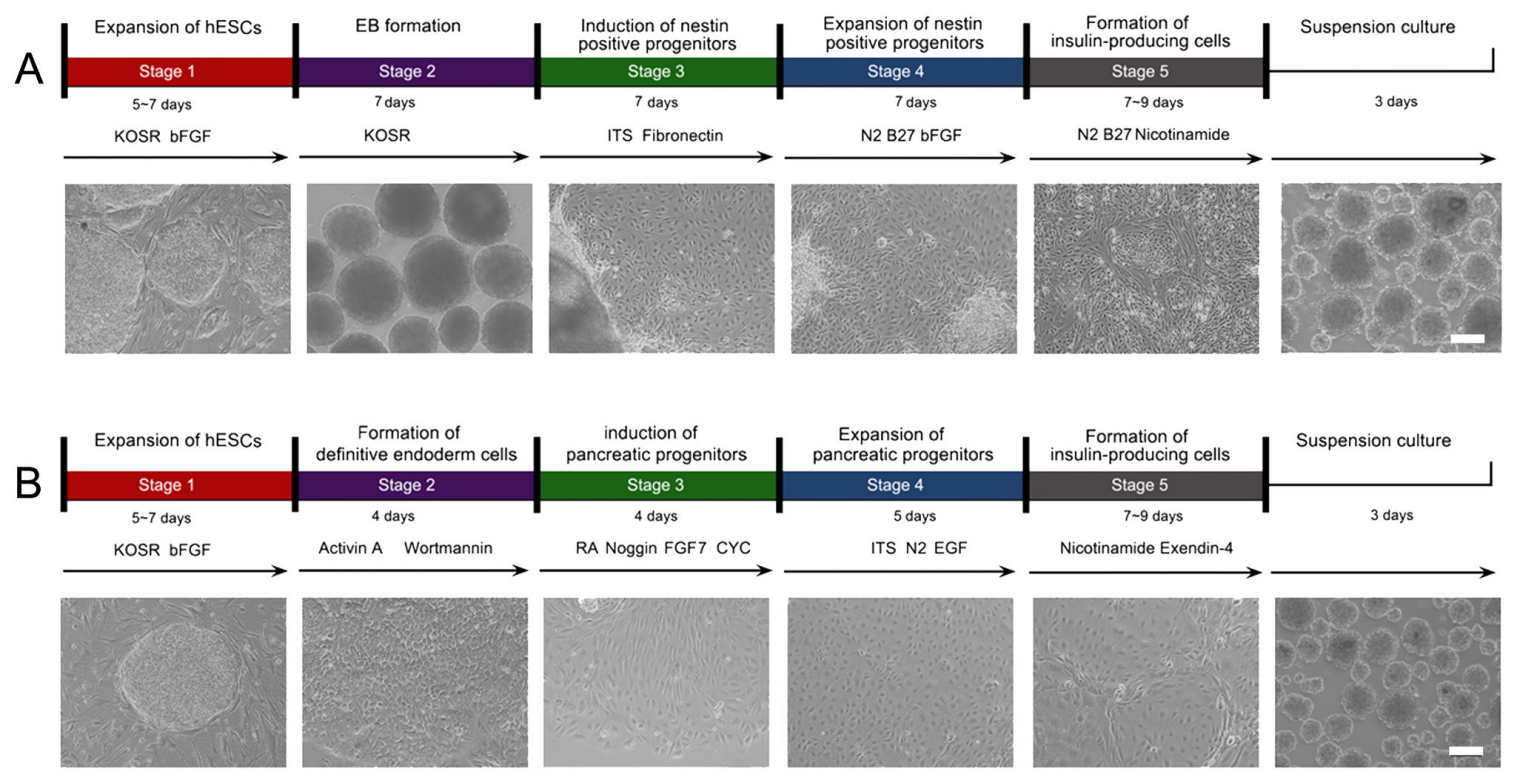
Differentiation of insulin-producing cells (IPCs) from hESCs. A: The upper panel displays the differentiation scheme for generating IPCs based on the selection of nestin-positive progenitors. The lower panel shows cell morphologies at different stages. B: The upper panel displays the differentiation scheme for generating IPCs through the definitive endoderm (DE) protocol. The lower panel shows the cell morphologies at different stages. Scale bars: 100 µm. hESCs: human embryonic stem cells; KOSR: KnockOut^TM^ serum replacement; EB: embryoid body; ITS: insulin-transferrin-selenium; bFGF: basic fibroblast growth factor; RA: retinoic acid; FGF 7: fibroblast growth factor 7; CYC: KAAD-cyclopamine; EGF: endothelial growth factor.

The five-stage nestin protocol for *in vitro* differentiation of hESCs into IPCs (Figure 1A) was modified from previously published protocols [4,5]:

Stage I: expansion of undifferentiated hESCs.

Stage II: formation of embryoid bodies (EBs). The hESCs were dissociated into small clumps by collagenase IV and were collected by sedimentation. The dissociated colonies were transferred to ultra-low attachment 6-well plates (Corning) and cultured for 7 days in differentiation medium in suspension. The differentiation medium consisted of 78% Dulbecco’s modified Eagle’s medium/F12 (DF12, Invitrogen) medium, 20% SR, 2 mM GlutaMAX, 1% NEAA and 0.1 mM β-mercaptoethanol.

Stage III: induction of nestin-positive progenitors. The EBs were cultured in DF12 medium supplemented with 1% insulin-transferrin-selenium (ITS, Invitrogen), 2 mM GlutaMAX and 5 µg/ml Fibronectin (Invitrogen) for 7 days.

Stage IV: expansion of nestin-positive progenitors. Stage III-cells were dissociated with 0.05% trypsin-EDTA (Invitrogen) and plated on plastic tissue-culture plates in DF12 medium supplemented with 1% N2 (Invitrogen), 2% B27 (Invitrogen), 2 mM GlutaMAX, and 10 ng/ml bFGF for 7 days. Before plating, the tissue-culture plates were coated with 0.1% gelatin (Sigma).

Stage V: formation of IPCs. Stage IV-cells were incubated in DF12 medium supplemented with 1% N2, 2% B27, 2 mM GlutaMAX, and 10 mM nicotinamide (Sigma) for 7-9 days. For further maturity, the cells were digested by 0.05% trypsin-EDTA and were transferred to ultra-low attachment 6-well plates for suspension cultures, then incubated for 3 days to form clusters.

The five-stage DE protocol for *in vitro* differentiation of hESCs into IPCs was modified from previously described protocols [6,8] and was performed as follows:

Stage I: expansion of undifferentiated hESCs.

Stage II: DE formation. The hESCs were dissociated into small clumps by collagenase IV and were collected by sedimentation. The dissociated colonies were plated on matrigel (1:50, BD Biosciences)-coated dishes (Corning) and incubated with DF12 supplemented with 100 ng/ml activin A (Peprotech) and 1 µM wortmannin (Sigma), 1% N2 and 1% B27 for 4 days.

Stage III: induction of pancreatic progenitor cells. Stage II-cells were cultured in IMDM/F12 with 2 µM retinoic acid (RA, Sigma), 20 ng/ml fibroblast growth factor 7 (FGF7, Peprotech), 50 ng/ml Noggin (Peprotech), 0.25 µM KAAD-cyclopamine (CYC, Calbiochem) and 1% B27 for 4 days.

Stage IV: expansion of pancreatic progenitor cells. Stage III-cells were cultured in DMEM (high glucose, Invitrogen) with 50 ng/ml endothelial growth factor (EGF, Peprotech), 1% ITS, and 1% N2 for 5 days.

Stage V: formation of IPCs. Stage IV-cells were incubated in DF12 (low glucose, Invitrogen) with 1% ITS, 10 ng/ml bFGF, 10 mM nicotinamide, 50 ng/ml exendin-4 (Sigma) for 7-9 days. For further maturity, the cells were digested by 0.05% trypsin-EDTA and were transferred to ultra-low attachment 6-well plates for 3-day suspension culture to form clusters.

### Real-Time Reverse Transcription Polymerase Chain Reaction (RT-PCR)

RNA samples were prepared from cells at different stages with an RNeasy Mini Kit (Qiagen) according to the manufacturer’s instructions. cDNA was synthesized from total RNA using reverse transcriptase by a First Strand cDNA synthesis Kit (Fermentas). Primers were designed using Primer 5.0 software. The cDNA was amplified by PCR using iQ^TM^ SYBR Green Supermix (BioRad) with an iQ5 real-time PCR detection system (Bio-Rad). All experiments were performed in triplicate. The sample input was normalized against the Ct (critical threshold) value of the housekeeping gene GAPDH. The expression level of each gene was normalized to the level of observed RNA isolated from cells differentiated using the nestin protocol, which was set as 100%. Primer sequences and PCR conditions used in this study are listed in Table 1.

**Table 1:** Primer sequence, annealing temperature, and product size of PCR reactions.

Genes	Primer sequence	Annealing temperature (°C)	Product size (bp)
Oct4	Forward: 5’-GGGTGGAGGAAGCTGACAAC-3’	60	114
	Reverse: 5’-GGTTGCCTCTCACTCGGTTC-3’		
Isl1	Forward: 5’-TGCAAGGACAAGAAGCGAAG-3’	62	91
	Reverse: 5’-GAGTTCCTGTCATCCCCTGG-3’		
Pdx1	Forward: 5’-CCCTCCTACAGCACTCCACC-3’	64	106
	Reverse: 5’-CCGCTGTGTGTGTTAGGGAG-3’		
MafA	Forward: 5’-TCATCCGGCTCAAGCAGAAG-3’	62	111
	Reverse: 5’-GTTGGCACTTCTCGCTCTCC-3’		
Insulin	Forward: 5’-CAGATCACTGTCCTTCTGCC-3’	62	105
	Reverse: 5’-GTTGGTTCACAAAGGCTGCG-3’		
GAPDH	Forward: 5’-TGCACCACCAACTGCTTAGC-3’	60	87
	Reverse: 5’-GGCATGGACTGTGGTCATGAG-3’		

For microRNA (miRNA) detection, total RNA samples were prepared from cells at different stages using Trizol (Invitrogen). Total RNA, including miRNA, was polyadenylated with poly (A) polymerase and reversely transcribed using a miRcute miRNA first-strand cDNA synthesis kit (Tiangen biotechnology, Beijing, China). The poly (A)-tailed cDNA was amplified with a miRcute (SYBR) miRNA qPCR detection kit (Tiangen) with an iQ5 real-time PCR detection system [24]. The sample input was normalized against the Ct value of the U6 gene. The forward primer sequences of miR-146a were miR-34a were from Tiangen. The other forward primer sequences synthesized by Beijing AuGCT DNA-SYN Biotechnology Company (China) were as follows: miR-7, 5’-CGGCGGTGGAAGACTAGTGATT-3’; miR-375, 5’-GCGTTTGTTCGTTCGGCTC-3’; and miR-145, 5’-GTCCAGTTTTCCCAGGAA-3’. The reverse primer sequence was universal (Tiangen). All annealing temperatures were 60^°^ C.

### Immunofluorescence

Cells that were grown in a monolayer were fixed for 20 min in 4% (w/v) paraformaldehyde in PBS at room temperature (RT), washed several times in PBS, and blocked for 30 min with 10% (v/v) normal serum (isotypic with the secondary antibodies) in PBS with 0.1% (v/v) Triton X-100 (Sigma). Primary and secondary antibodies were diluted in PBS. Primary antibodies were incubated for 24 h at 4^°^ C, and secondary antibodies were incubated for 1 h at RT, followed by washing and staining with DAPI. Images were captured under a fluorescent microscope (Nikon). Negative controls were performed by using corresponding isotypic sera to replace the primary antibodies. The following antibodies and dilutions were used: rabbit anti-Sox9 (Santa Cruz), 1:200; mouse anti- fetal liver kinase-1 (Flk1, Santa Cruz), 1:100; rabbit anti-nestin (Boster Bioengineering, Wuhan, China), 1:200; mouse anti-β III tubulin (Santa Cruz), 1:400; mouse anti-Foxa2/Hnf 3β (Santa Cruz), 1:400; goat anti-Sox17 (R&D Systems), 10 µg/ml; rabbit anti-Hnf1β (Santa Cruz), 1:400; goat anti-Nkx6.1 (Santa Cruz), 1:200; goat anti-pancreatic and duodenal homeobox 1 (Pdx1, Santa Cruz), 1:400; mouse anti-insulin (Sigma), 1:1,000; rabbit anti-C-peptide (Cell signaling), 1:200; rabbit anti-pancreatic polypeptide (PP, Chemicon), 1:200; mouse anti-glucagon (Sigma), 1:500. FITC- or TRITC-conjugated goat anti-rabbit IgG, FITC- or TRITC-conjugated goat anti-mouse IgG, and TRITC-conjugated rabbit anti-goat IgG (Beijing Zhongshan Biotechnology, China) were used at 1:200 dilutions.

### Insulin release

Stage V-clusters in suspension culture were rinsed twice in Krebs-Ringer Bicarbonate HEPES (KRBH) buffer (120 mM NaCl, 5 mM KCl, 2.5 mM CaCl_2_, 1.1 mM NaHCO_3_, 0.5% bovine serum albumin, and 10 mM HEPES) and preincubated for 1 h in KRBH buffer. The clusters were then incubated for 1 h in KRBH buffer with either 2.5 (low level) or 16.7 mM (high level) glucose at 37^°^ C. The medium was retrieved and stored at -20^°^ C. To measure protein content, the cells were harvested and homogenized by sonication in water, then stored at -80^°^ C. Insulin levels were measured using a Human Insulin ELISA Kit (Millipore). Protein concentrations were determined using a BCA^TM^ Protein Assay Kit (Thermo Scientific).

### Flow cytometry

Cells were harvested and washed twice in cold Hanks’ Balanced Salt Solution (HBSS). A total of 5×10^5^ cells in cold 2% paraformaldehyde fixative were resuspended and incubated at RT for 10 min. The cells were washed twice in HBSS and resuspended in 200 µl of 0.5% (w/v) Tween in PBS, then 10 µl APC (allophycocyanin)-conjugated anti-insulin antibody (R&D system) were added and the cells were incubated for 40 min at RT in the dark. The cells were washed twice in Tween buffer, then resuspended in 300 µl PBS for final flow cytometric analysis. Isotypic sera were used to replace the primary antibodies as the negative control, and the mouse β cell line Min6 at passage 52 (P52 Min6) was used as the positive control.

### Statistical analysis

Data are expressed as mean ± SD. Statistical analysis was assessed by SPSS statistical package (SYSTAT Software Inc). Comparisons between groups were carried out with a student’s t test or one-way ANOVA tests when appropriate. A *P* value < 0.05 was considered statistically significant. 

## Results

### hESC differentiation into IPCs using the nestin protocol

**Figure 2:**
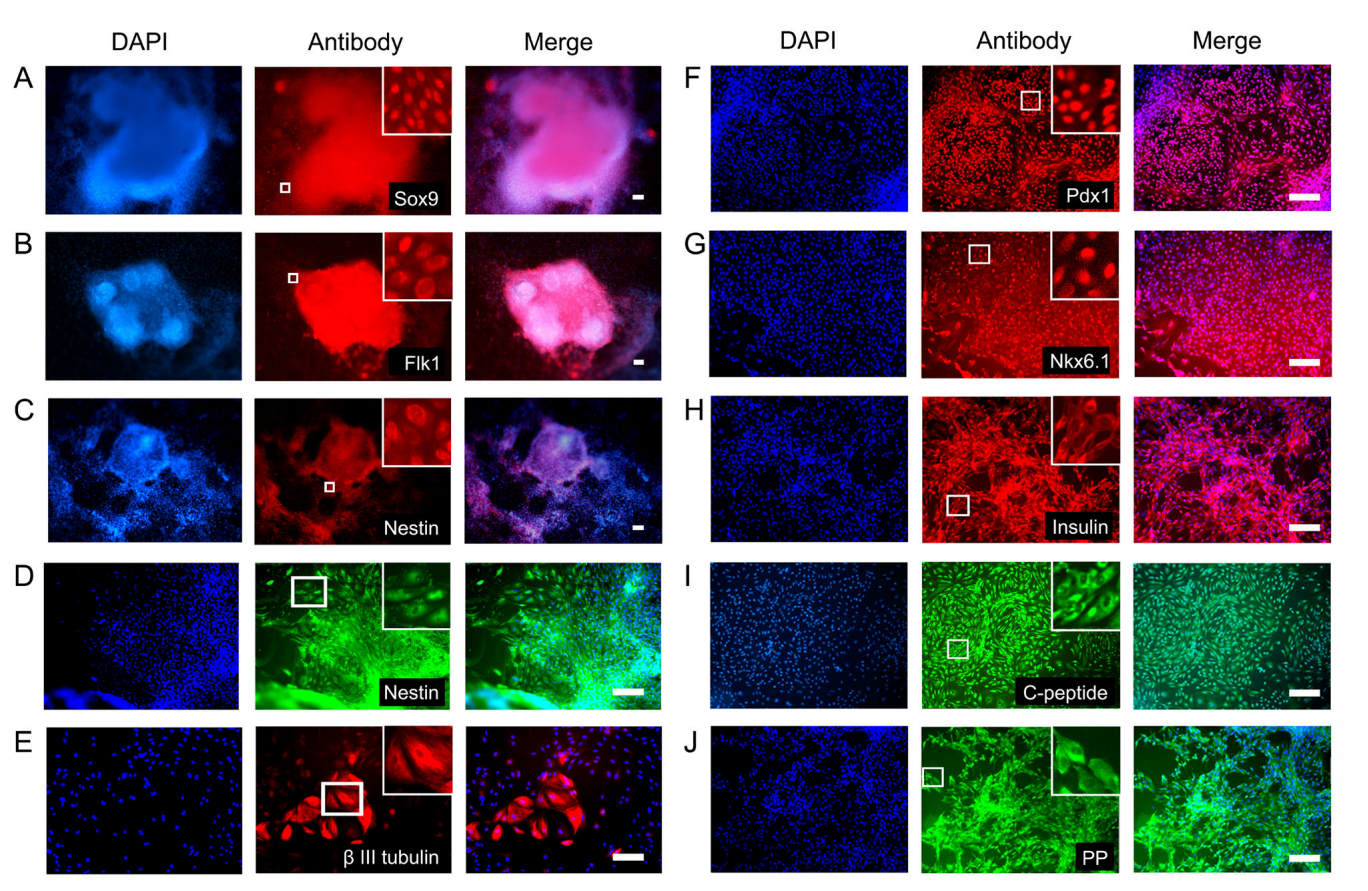
Immunofluorescent analysis of specific markers expressed at different stages using the nestin protocol. A–C: An embroid body (EB) at stage II; D: Pancreatic progenitors at stage III; F-G: Pancreatic progenitors at stage IV; E, H–J: IPCs at stage V. Left lane: DAPI staining; middle lane: specific markers; right lane: merged. The zoom-in boxes show an enlarged field for each group. Scale bars: 100 µm. Pdx1, pancreatic and duodenal homeobox 1; PP, pancreatic polypeptide.

**Figure 3:**
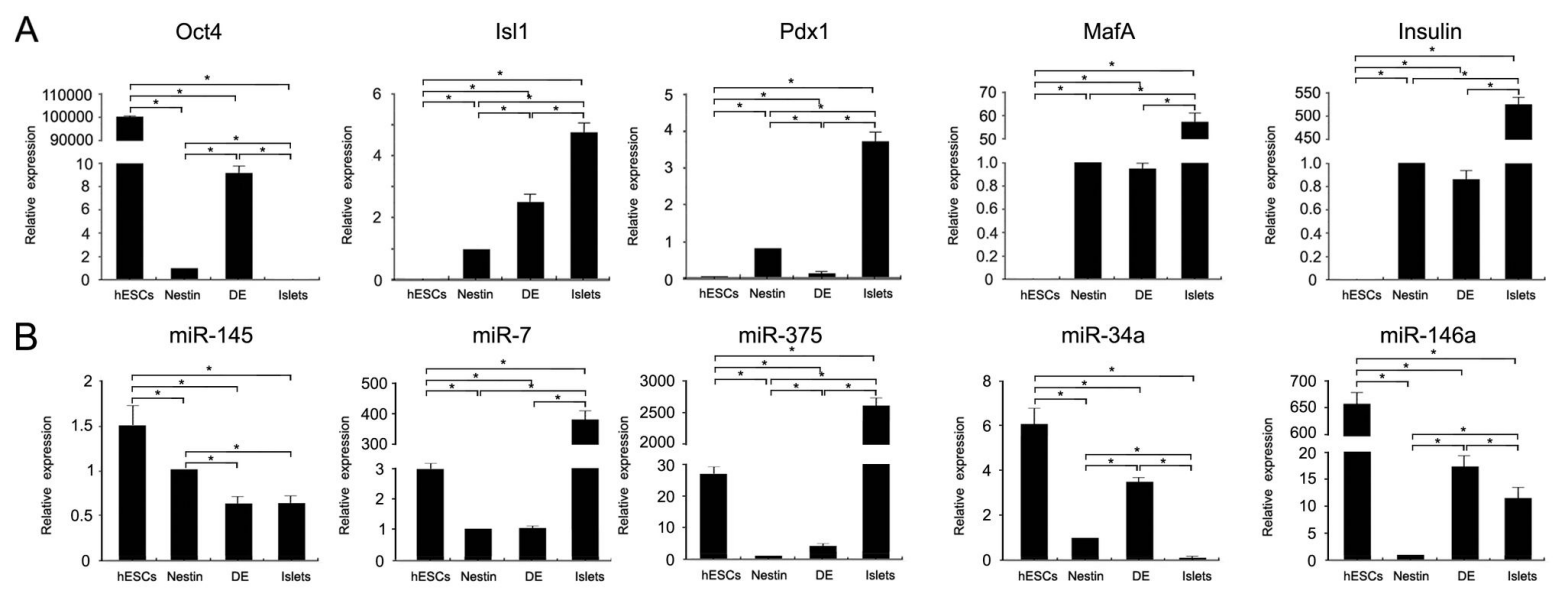
Real-time PCR analysis of gene expression. Comparison of relative mRNA (A) and miRNA (B) expression in IPCs differentiated from the nestin and DE protocols as determined by real-time PCR analysis. hESCs and adult human islets served as controls. The expression level in cells from the nestin protocol was set at 100%. All experiments were performed in triplicate in at least four separate experiments. Statistical analysis was assessed by one-way ANOVA tests. Differences between different groups are shown in the figure. A *P*<0.05 was considered statistically significant. * *P*<0.05.

The induction of hESC differentiation into IPCs was documented at different stages. The nestin-positive progenitor selection strategy relied on initial EB formation (Figure 1A, second column). EBs were the spherical arrangements of ESCs destined to differentiate into progenitors of all three germinal layers, including endoderm (Sox9+), mesoderm (Flk1+), and ectoderm (nestin+) (Figure 2A–C, Supplemental Figure S1). Sequential treatment of EBs with a cocktail of growth factors enforced a lineage commitment pathway that initially gave rise to nestin-positive cells (Figure 1A, third column and Figure 2D), which subsequently differentiated into endocrine progenitors (Figure 1A, fourth column), and ultimately, into IPCs (Figure 1A, fifth column). The IPCs in the suspension culture aggregated into islet-like clusters (Figure 1A, sixth column and Supplemental Figure S2A, first column). To characterize the cells, we examined gene expression at the protein level using immunofluorescence. Well characterized transcription factors of pancreatic progenitor cells (Nkx6.1 and Pdx1) at stage IV, and pancreatic hormones (insulin, C-peptide and PP) at stage V were detected in the progenitor cells and IPCs, respectively. Their positive percentage reached approximately 90% (Figure 2F–J). The mRNA expression of Pdx1 and insulin was also found by quantitative RT-PCR (Figure 3A), suggesting pancreatic specialization and IPC formation. Taken together, the specific expression patterns of these pancreatic hormones and transcription factors strongly indicated that IPCs had been obtained using the nestin-positive progenitor cell protocol.

### hESCs were successfully induced to differentiate into IPCs using the DE protocol

**Figure 4:**
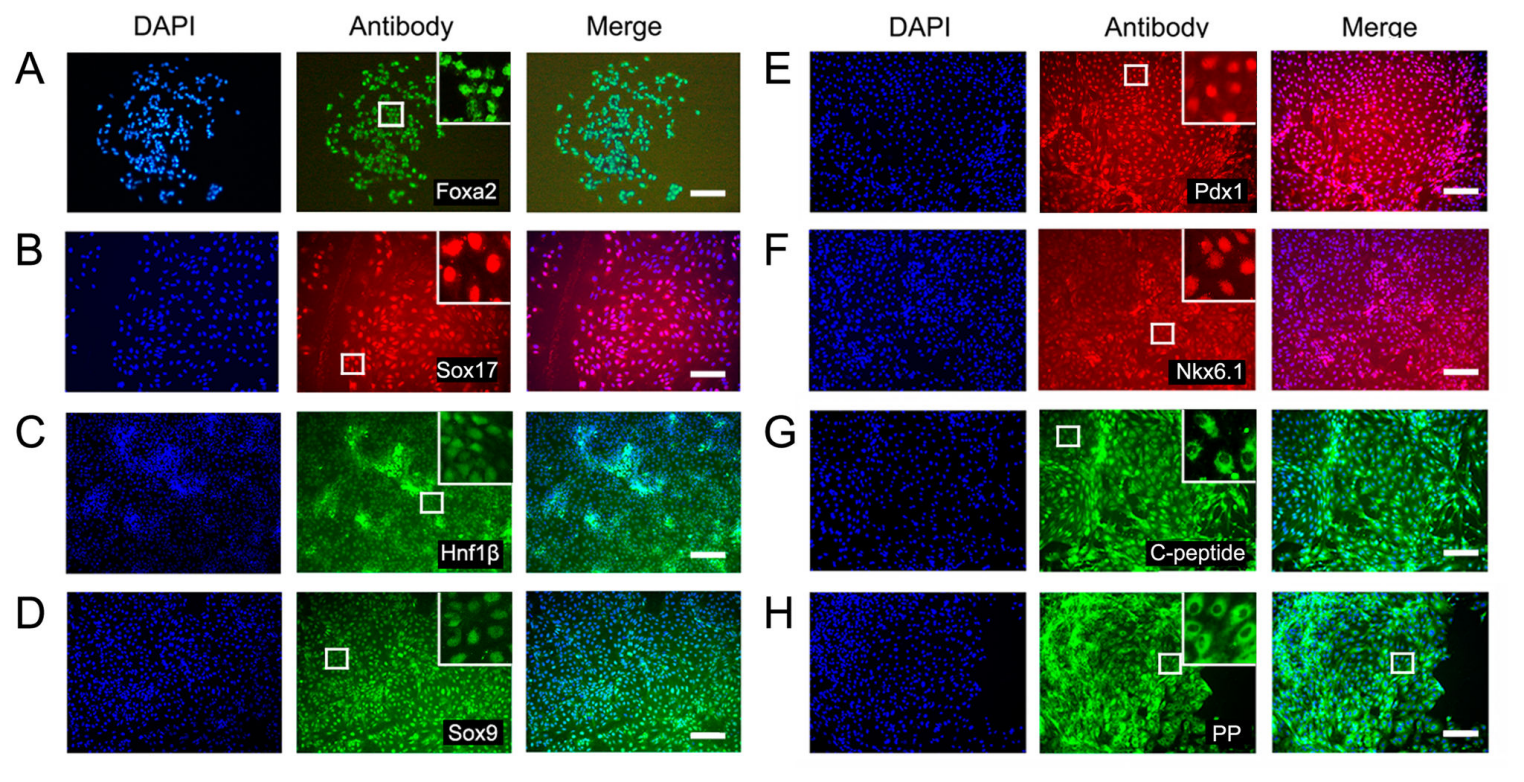
Immunofluorescent analysis of specific markers expressed at different stages in the DE protocol. A–B: DE at stage II; C–D: Pancreatic progenitors at stage III; E-F: Pancreatic progenitors at stage IV; G-H: IPCs at stage V. Left lane: DAPI staining; middle lane: specific markers; right lane: merged. The zoom-in boxes show an enlarged field in each group. Scale bars: 100 µm. Pdx1, pancreatic and duodenal homeobox 1; PP, pancreatic polypeptide.

Differentiation of hESCs into IPCs was also achieved by bypassing EB formation and selectively generating DE from which all gastrointestinal organs originate (Figure 1B). With this approach, hESCs were treated with activin A and the PI3K (phosphatidylinositol 3-kinase) inhibitor wortmannin. Cells expressing Sox17 and Foxa2 were detected by immunofluorescence at stage II (Figure 1B, second column and Figure 4A-B). Subsequently, activation of FGF and RA signaling combined with inhibition of BMP (bone morphogenetic protein) and SHH (sonic hedgehog) signaling led the DE cells towards a pancreatic fate (Figure 1B, the third column). The gut-endoderm specific markers Hnf1β and Sox9 were detected in the stage III cells (Figure 4C-D), and the pancreatic progenitor markers Pdx1 and Nkx6.1 were visible in the stage IV cells (Figure 4E-F). Further treatment of the differentiated cells with a cocktail of growth factors led to the generation of IPCs (Figure 1B, fifth column), as indicated by the expression of the pancreatic hormones C-peptide and PP (Figure 4G-H) and the transcription factors Isl1, Pdx1, and MafA (Figure 3A) in the stage V cells.

### Similarities between the nestin and DE protocols for IPC differentiation of hESCs

**Figure 5:**
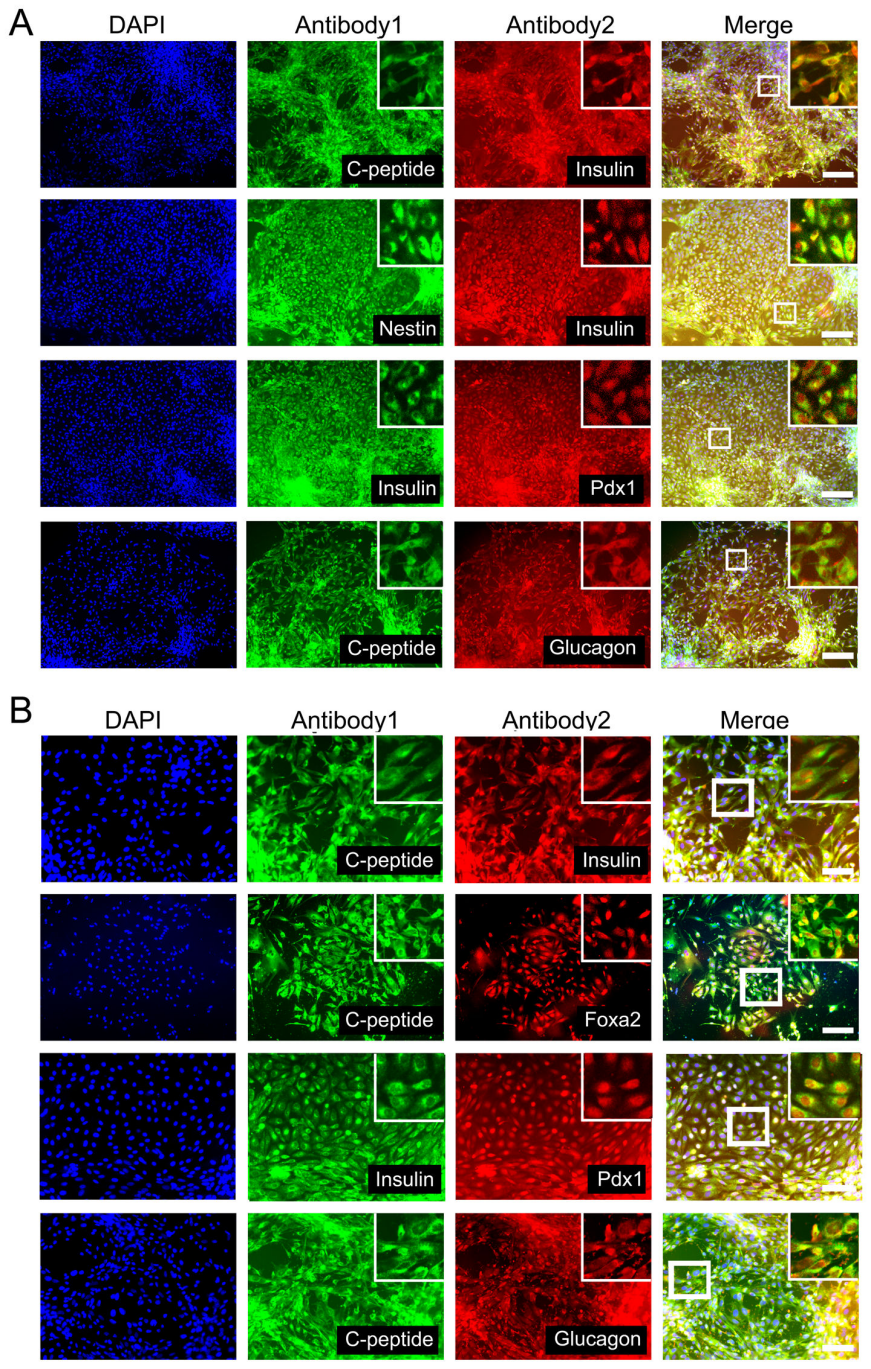
Double-labeling immunofluorescence of IPCs at stage V. A: IPCs differentiated with the nestin protocol. Scale bars: 100 µm. B: IPCs differentiated with the DE protocol. Scale bars: 50 µm. Left lane: DAPI staining; middle lanes: specific markers; right lane: merged. The zoom-in boxes show an enlarged field for each group. Pdx1, pancreatic and duodenal homeobox 1.

**Figure 6:**
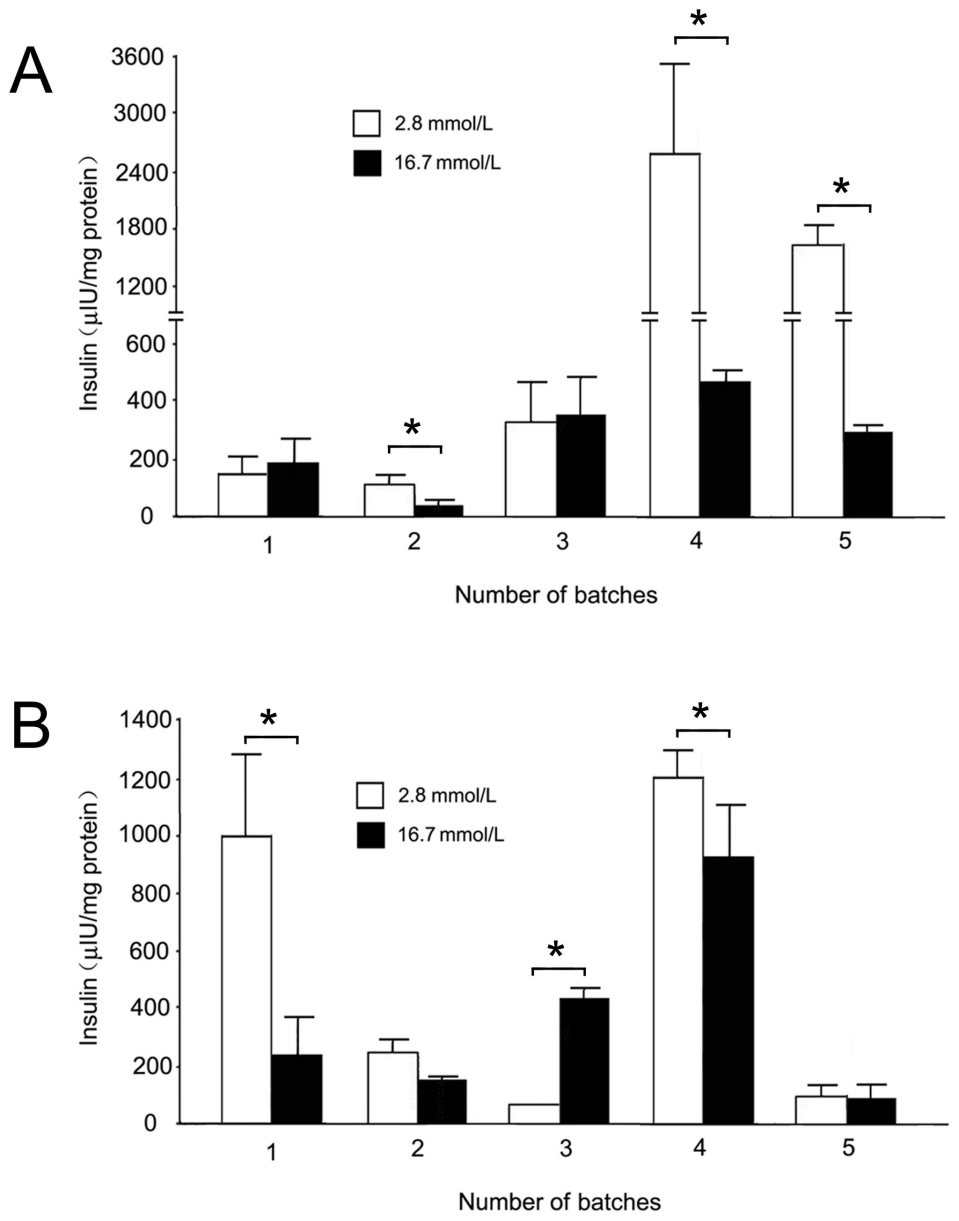
The glucose-challenged insulin release response in stage V-cell clusters. A: IPCs differentiated using the nestin protocol. B: IPCs differentiated using the DE protocol. Each study batch was analyzed in at least three independent experiments. Data are presented as mean ± SD. Statistical analysis was assessed by the Student’s t test. A *P*<0.05 was considered statistically significant. * *P*<0.05.

hESC differentiation into IPCs was successfully induced with both protocols, and the cells from both protocols shared several characteristics. First, stage V cells in suspension culture were able to aggregate into islet-like clusters which could be stained by dithizone (Figure 1, sixth columns and Figure S2). The expression of key transcription factors for pancreatic progenitor cells (Pdx1 and Nkx6.1) at stage IV and pancreatic hormones (insulin, C-peptide and PP) at stage V were detectable in cells derived from both protocols (Figures 2F–J and 4E–H). Co-immunostaining of Pdx1 with insulin suggested that the IPCs were differentiated from the Pdx1 positive pancreatic progenitor cells (Figure 5). Co-expression of insulin and C-peptide validated *de novo* insulin synthesis rather than uptake of insulin from the culture media (Figure 5). Furthermore, C-peptide and glucagon produced by different pancreatic endocrine cell types were co-immunostained with each other in the stage V-cells (Figure 5). One of the most important functions of pancreatic islets is their insulin secretion. Therefore, we tested the glucose-challenged insulin release response in the IPCs. Cells derived from both protocols showed poor insulin release in response to glucose, and the levels of insulin release varied between different batches (Figure 6 and table S1). This observation was reminiscent of the immature cells’ characteristics generated by both protocols. In support of this inference, the differential expression of several genes was found when the IPCs were compared with adult human islets (Figure 3). The expression levels of insulin and transcription factor MafA in the differentiated cells were extremely lower than that of the human adult islets, whereas the expression levels of Isl1 and Pdx1 were similar to those observed in the islets. When compared with hESCs, however, the IPCs showed much higher expression levels of pancreatic hormone and transcription factors, and the hESC-specific marker Oct4 levels were dramatically decreased and nearly reached nadir. This suggested that efficient differentiation had taken place.

### Differences between the nestin and DE protocols for IPC differentiation of hESCs

We observed many differences between the two differentiation protocols. Both protocols resulted in different differentiation stages which showed specific marker expression profiles. In the nestin protocol, the cell population first formed EBs, which contained all three germinal lineages including nestin-positive ectoderm (Figure 2A–C). With the selection of nestin-positive progenitors, this population was amplified and further differentiated into IPCs. β III tubulin expression was observed in the terminal differentiation stage-cells, which further verified the presence of ectoderm derivatives (Figure 2E). In the DE protocol, the cell population progressed through the differentiation stages in concordance with pancreatic development, including the induction of Foxa2+ Sox17+ DE (Figure 4A-B), the formation of Hnf1β+ Sox9+ gut-tube endoderm (Figure 4C-D), the specification of Pdx1+ Nkx6.1+ pancreatic precursors (Figure 4E-F), and the final maturation into IPCs (Figure 4G-H). At stage V, insulin was co-expressed with nestin in the IPCs of the nestin protocol, whereas C-peptide co-immunostained with Foxa2 in the differentiated cells of the DE protocol (Figure 5). This indicated that the IPCs were derived from the ectoderm and endoderm derivatives, respectively.

The mRNA expression levels of specific markers determined by quantitative RT-PCR were not identical in the IPCs derived from both protocols (Figure 3A). The hESC-specific marker Oct4 expression level was 9.37-fold higher in cells from the DE protocol when compared with those differentiated using the nestin protocol. Progenitor marker Isl1 level was 2.45-fold higher in the DE protocol cells than that in the nestin protocol cells, while Pdx1 expression level in the DE protocol cells was less than a tenth of the expression recorded in the nestin protocol cells. Insulin and maturation maker MafA expression in the IPCs were comparable between the two groups.

miRNAs are small non-coding RNAs that play critical roles in post-transcriptional regulation of gene expression. miRNAs participate in pancreatic development [25–29] and modulate specific differentiation of ESCs [7,30,31]. In the present study, the expression of the miRNAs associated with islet development and function was analyzed. The levels of miR-145, miR-7, miR-375, miR-34a, and miR-146a were 0.61, 1.02, 4.07, 3.47, and 17.39 fold, respectively, in the IPCs of the DE protocol when compared with those of the nestin protocol (Figure 3B). These results suggest that epigenetic regulation is different when either protocol is used.

**Figure 7:**
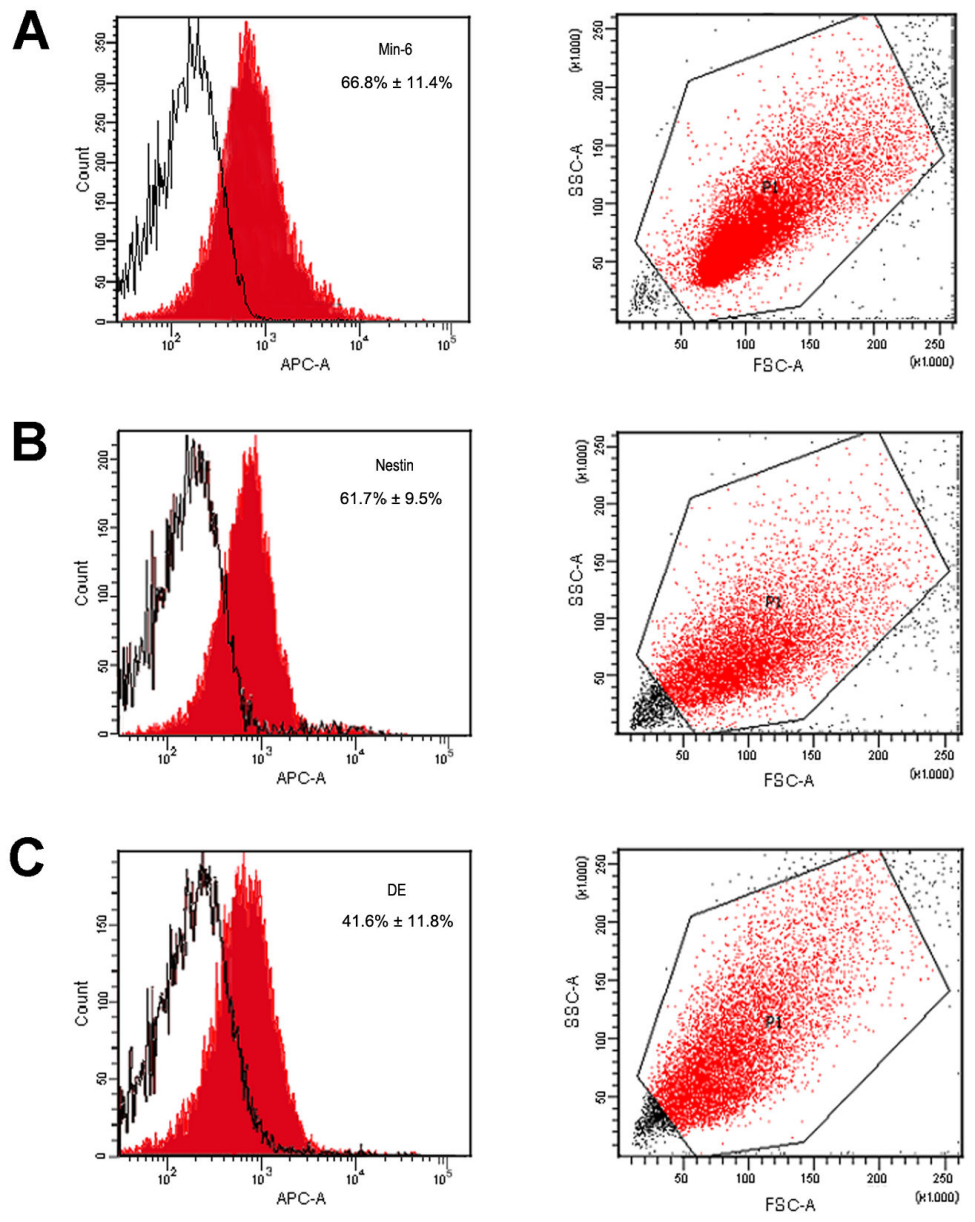
Flow cytometry analysis of stage V-cells from both protocols. A: The mouse pancreatic islet β cell line Min6 served as the positive control. B: Differentiated IPCs using the nestin protocol. C: Differentiated IPCs using the DE protocol. The left lane shows cells detected with an anti-insulin antibody. The positive rates were 66.8% ± 11.4%, 61.7% ± 9.5%, and 41.6% ± 11.8% for the P52 Min6 cells, the nestin protocol cells, and the DE protocol cells, respectively. Each study batch was analyzed in at least three independent experiments. Data are presented as mean ± SD. The right lane shows the scatter plot manifested by the forward scatter (FSC) and side scatter (SSC).

Cell morphology from both protocols at different stages showed specific traits. At the end of differentiation, typical endocrine cells from the nestin protocol were in multilayered regions and nested structure; while the endocrine cells from the DE protocol were mostly found in monolayer and showed classic epithelium-like characteristics (Figure 1, fifth columns). The scatter plots show that the two differently derived cell populations varied in size and granularity as detected by forward scatter (FSC) and side scatter (SSC) flow cytometry analysis (Figure 7). Moreover, IPC yields differed between the two protocols. Assessment of IPC quantity by flow cytometry revealed that the differentiation cultures produced an average of 61.7% ± 9.5% and 41.6% ± 11.8% insulin-positive cells in the nestin and DE protocols, respectively (Figure 7). A 100 mm plate with 10^6^ undifferentiated hESCs yielded ~10^8^ and 5×10^7^ cells at stage V in the nestin and DE protocols, respectively. Therefore, the average yields of insulin-positive cells in the two protocols were ~6×10^7^ and 2×10^7^ from 10^6^ hESCs. Lastly, the duration of the two differentiation protocols was quite different. The nestin protocol required 28 days to obtain IPCs, whereas 20 days were needed for the DE protocol. 

## Discussion

**Figure 8:**
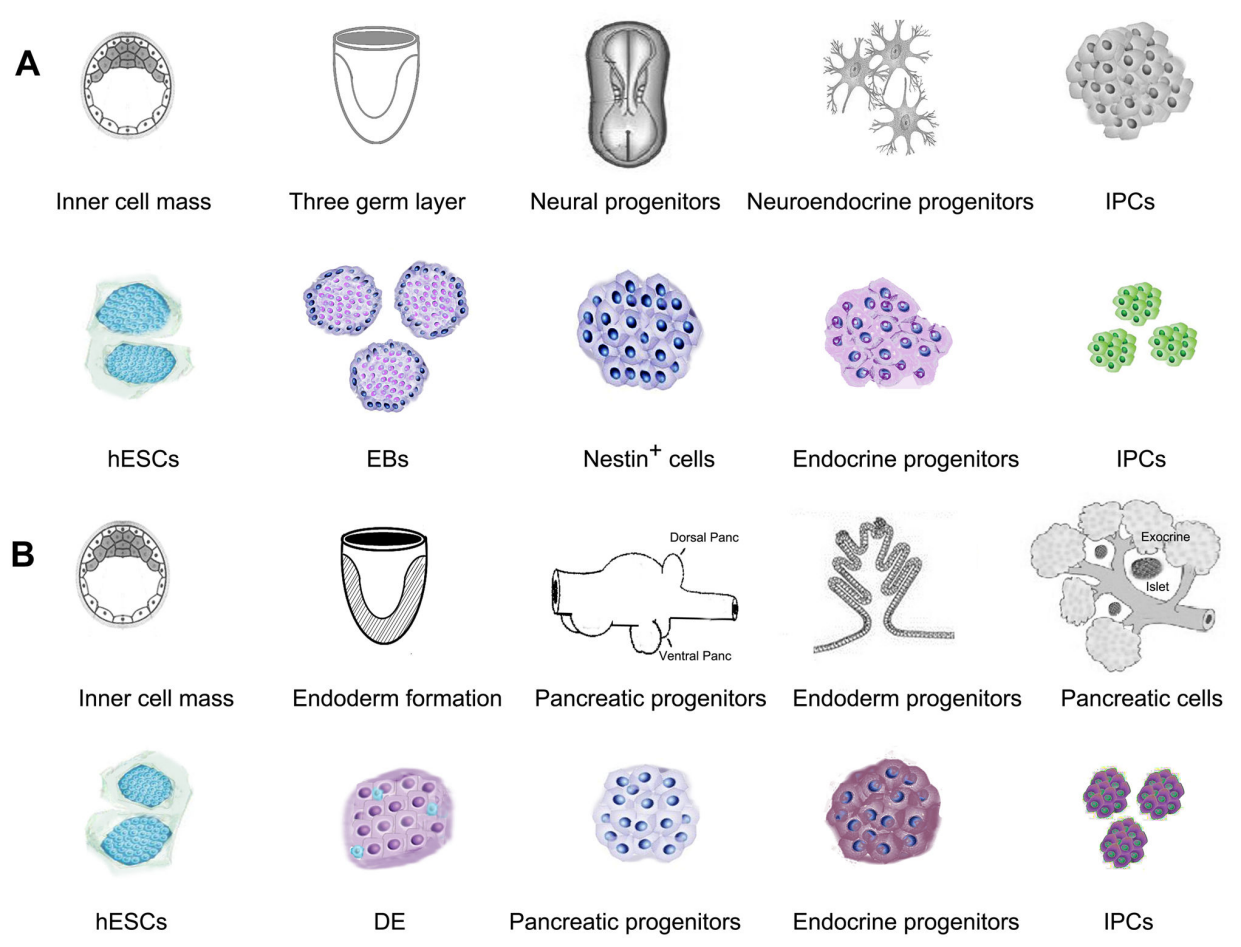
The lineage of the developing pancreas *in vivo* and the IPC differentiation of hESCs *in vitro*. A: The upper panel displays a possible pathway for IPCs during neural development. The lower panel shows the lineage of the developing IPCs using the nestin protocol. B: The upper panel displays the pancreatic morphogenesis at different stages of development. The lower panel shows the lineage of the developing IPCs using the DE protocol. Schematics in Figure 8B are modified from a previous review [38].

Developing alternative ways to restore pancreatic β cell mass in patients with diabetes is a challenging problem. hESCs provide an alternative cell source for the regenerative medicine in diabetes as they are characterized by unlimited self-renewal and the ability to differentiate into IPCs. So far, there are two main approaches for the differentiation of hESCs into IPCs (Figure 1 and Figure 8). One is based on the selection of nestin-positive progenitor cells through EB formation [4,5], and the other is to acquire pancreatic progenitor cells by inducing the generation of DE [6,8]. Although protocols can successfully be used to differentiate hESCs into IPCs, it is still debated which protocol is better suited for future use in the treatment of diabetes. In recent years, more studies have supported the viewpoint that hESC-derived endoderm and pancreatic lineages may have better therapeutic potential. However, this is only theoretical supposition, and there is no evidence for direct comparison between the data obtained from these two protocols. In addition, each of the hESC cell lines exhibits a marked propensity to differentiate into the specific lineages, often with > 100-fold differences in lineage-specific gene expression [19]. Therefore, it is necessary to analyze the differences of these two protocols in the same hESC cell line for pancreatic β cell generation.

Our results show that there are many similarities between the nestin and DE protocols, such as IPC morphology in suspension culture, the expression of marker transcription factors and pancreatic hormones, polyhormonal expression, and poor insulin release in response to glucose. On the other hand, there are important differences between these two differentiation strategies, such as specific marker expression profiles at different stages, expression levels of pancreas-specific markers, and sizes, granularities, and yields of resulting IPCs. These results indicate that the IPCs generated by the two protocols have their own specific gene expression profiles although they both, at least in part, share several characteristics with human pancreatic islets.

The major difference is that each protocol is based on a different differentiation theory. The nestin protocol is based on the similarities observed between pancreatic β cell and neuroepithelial development [14,16]. It has been proposed that nestin, a neuroﬁlament protein marker of neuronal progenitors, is expressed in human pancreatic β cell precursors [17]. Therefore, this protocol can induce differentiation of ESCs into IPCs by selecting for nestin-positive cells [4,5,9,18]. In a word, the nestin protocol is similar to that of neural development before trans-differentiating to IPCs (Figure 8A). In contrast, the DE protocol may be explained by the recapitulation of islet organogenesis during fetal pancreatic development *in vivo* (Figure 8B). Therefore, the IPCs derived from this path are more similar to the authentic pancreatic β cells. It is also worthwhile to note that this protocol reproduces the formation of pancreatic dorsal anlage.

Ventral and dorsal pancreata develop from anterior and posterior foregut endoderm respectively, and each may require different signals from the mesenchyme. Moreover, the endocrine cell development appears to occur in two waves [32]. Differences in the origin of dorsal and ventral pancreata and their required signaling are indicative of the disparity in initial developmental programs and further maturation. Rodent and human pancreatic islet β cells are derived from more than one progenitor [13,33], suggesting that pancreatic islets are heterogeneous. Although pancreatic β cells are the main source of insulin production in mammals, they are not the only cell type that can synthesize and release insulin. Other IPCs can be found in the yolk sac, fetal liver, and certain neuronal cell types [34]. In addition to insulin, these extra-pancreatic cell types also express several other genes in common with true β cells.

The nestin protocol is based on the similarities between pancreatic β cell and neuroepithelial development [4,5,9,18]. It may be that these two closely related tissues are interconvertible. Adult hepatocytes, pancreatic exocrine cells, and pancreatic endocrine α cells are able to successfully trans-differentiate into insulin-producing β cells [35–37], but it often involves virus particle-mediated gene induction. However, the supplementation of growth factors, cytokines, small molecules, and matrix may be also feasible. Our results show that IPCs in suspension culture can aggregate into islet-like clusters and express pancreas-specific markers including pancreatic hormones and key pancreatic transcription factors at stage V of the nestin protocol. These data suggest that long-term cultures of nestin-positive pancreatic progenitor cells may be considered as a potential source in generating functional β-like cells (Figure 8A).

Despite improvements in currently available differentiation protocols of hESCs into IPCs, most methods are labor intensive, expensive, and time consuming. The resulting IPCs are still immature, and the amount of insulin production from each IPC cluster appears to be less than a tenth of that produced by a single human isolated islet in our preliminary experiments (data not shown). Moreover, the molecular mechanisms of pancreatic islet β cell development, especially in the signaling pathways that instruct endocrine progenitor cells to differentiate into mature and functional β cells, are poorly understood. For the hESC-derived IPCs to become a potential cell source for clinical use in diabetes therapy, a novel differentiation protocol with significant improvement needs to be developed and the mechanism of pancreatic islet differentiation deserves further investigation.

In conclusion, the differentiation of hESCs into IPCs creates a possible source for cell replacement therapy. Both the nestin and DE protocols have produced IPCs that have molecular characteristics which closely resemble bona fide insulin-secreting β cells. However, the expression levels of pancreatic islet-specific markers in these IPCs were much lower than those of adult human islets. Moreover, several pancreatic hormones were co-expressed in the IPCs, yet these cells were often unresponsive to glucose. In these aspects, the two differentiation strategies shared many similarities. On the other hand, the IPCs derived from different origins showed protocol-specific expression profiles at different stages. Analyzing the similarities and differences between these two protocols will help us to realize the distinct advantages and disadvantages of each differentiation process, and may be beneficial in drawing lessons from one process to be applied to the other. Despite the significant progress made, numerous challenges still need to be overcome before either differentiation protocol can be exploited for large scale of high-quality IPCs.

## Supporting Information

Figure S1 **Immunofluorescent analysis of specific markers of embroid body performed in cross-sections.** Left lane: DAPI staining; middle lane: specific markers; right lane: merged. The zoom-in boxes show an enlarged field in each group. Scale bars: 100 µm.
(TIF)

Figure S2 **Cells in suspension culture with dithizone (DTZ) staining.** A: Cell morphologies in suspension culture. B: The DTZ staining. Images from left to right are: IPCs in suspension culture from the nestin protocol, IPCs in suspension culture from the DE protocol, primary rat islets, and embroid bodies (EBs). Scale bars: 100 µm.
(TIF)

Table S1 **The glucose-challenged insulin release response in stage V cell clusters.**
(DOC)
