# Supplementary figures and images for: Insulin-Producing Cells Derived from Human Embryonic Stem Cells: Comparison of Definitive Endoderm- and Nestin-Positive Progenitor-Based Differentiation Strategies
Source: PLoS One. 2013 Aug 12;8(8):e72513. doi: 10.1371/journal.pone.0072513 (PMC3741181; doi:10.1371/journal.pone.0072513)

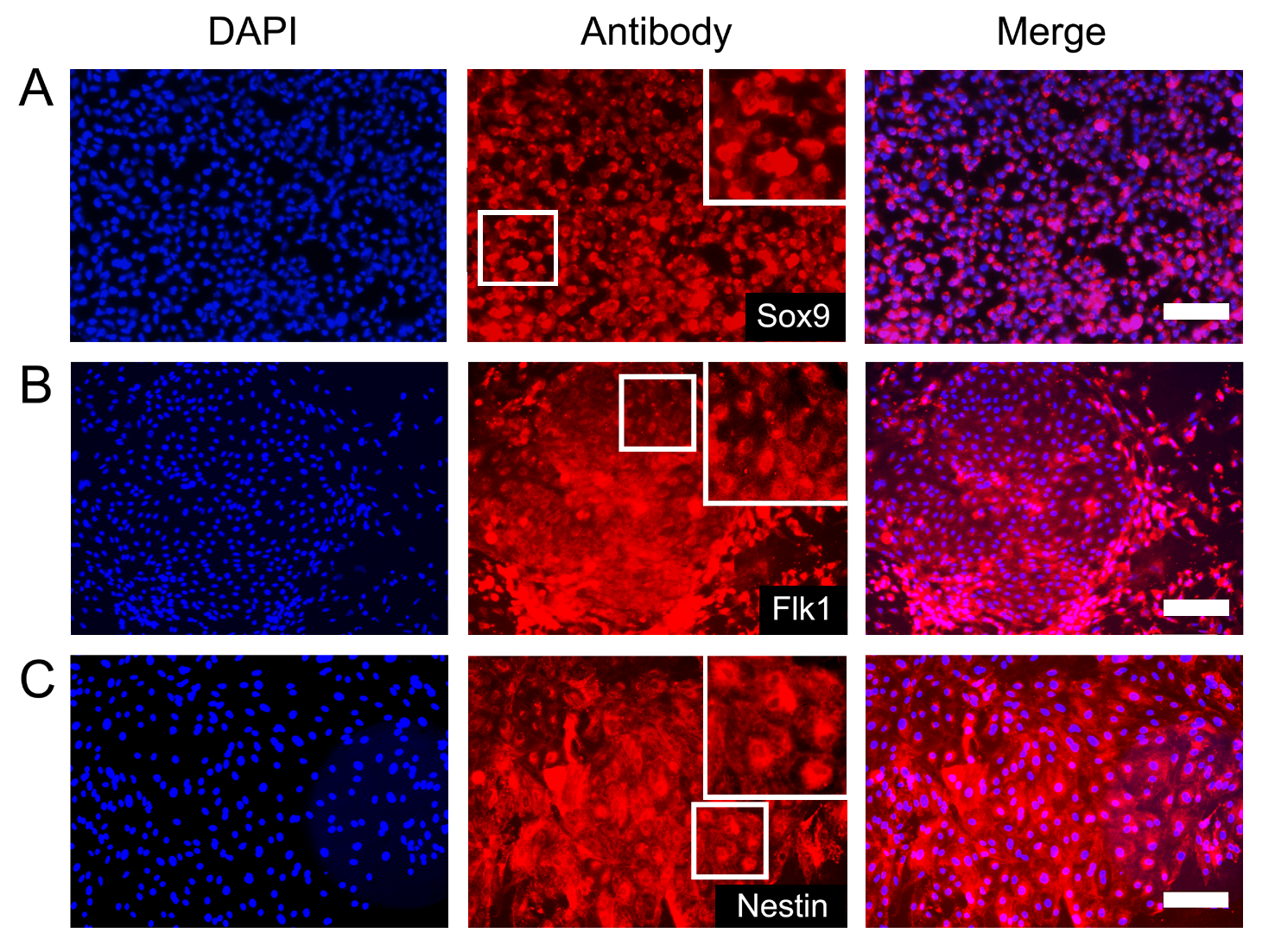

Supplement: Figure S1 — Immunofluorescent analysis of specific markers of embroid body performed in cross-sections. Left lane: DAPI staining; middle lane: specific markers; right lane: merged. The zoom-in boxes show an enlarged field in each group. Scale bars: 100 µm. (TIF) [file pone.0072513.s001.tif]

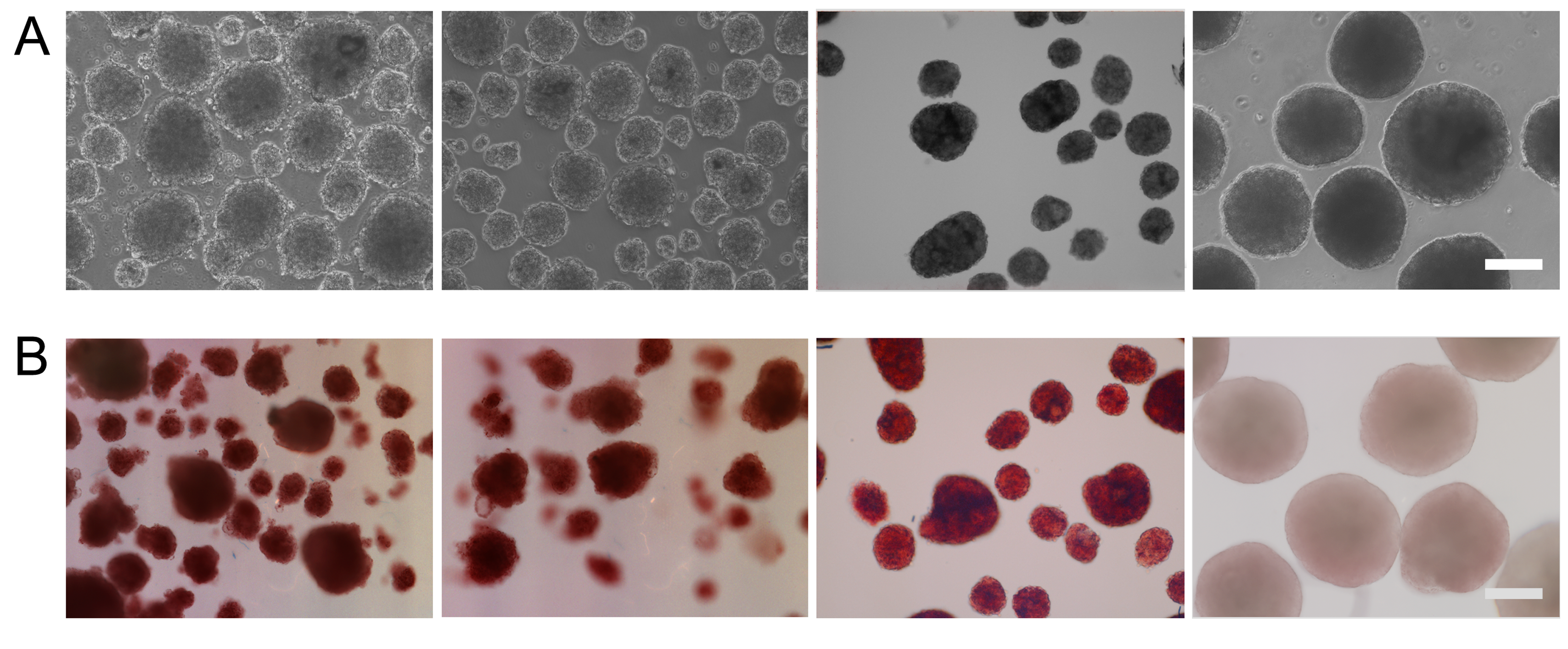

Supplement: Figure S2 — Cells in suspension culture with dithizone (DTZ) staining. A: Cell morphologies in suspension culture. B: The DTZ staining. Images from left to right are: IPCs in suspension culture from the nestin protocol, IPCs in suspension culture from the DE protocol, primary rat islets, and embroid bodies (EBs). Scale bars: 100 µm. (TIF) [file pone.0072513.s002.tif]
