# Supplementary material for: Insulin-Producing Cells Derived from Human Embryonic Stem Cells: Comparison of Definitive Endoderm- and Nestin-Positive Progenitor-Based Differentiation Strategies
Source: PLoS One. 2013 Aug 12;8(8):e72513. doi: 10.1371/journal.pone.0072513 (PMC3741181; doi:10.1371/journal.pone.0072513)
Supplement: Table S1 — The glucose-challenged insulin release response in stage V cell clusters. (DOC) [file pone.0072513.s003.doc]

**Supplemental table S1.** The glucose-challenged insulin release response in stage V cell clusters.

| Batch | Insulin release in low glucose (μIU/mg protein) | Insulin release in high glucose (μIU/mg protein) | *P* |
| --- | --- | --- | --- |
| Nestin protocol | | | |
| No. 1 | 143.7 ± 57.3 | 180.4 ± 75.0 | 0.538 |
| No. 2 | 106.2 ± 34.2 | 37.0 ± 17.2 | 0.011 |
| No. 3 | 321.2 ± 140.4 | 340.3 ± 124.4 | 0.868 |
| No. 4 | 2609.3 ± 974.8 | 457.7 ± 34.0 | <0.001 |
| No. 5 | 1599.3 ± 234.7 | 249.4 ± 19.9 | <0.001 |
| DE protocol | | | |
| No. 1 | 991.9 ± 284.9 | 229.4 ± 137.8 | 0.009 |
| No. 2 | 239.6 ± 38.8 | 137.4 ± 13.6 | 0.072 |
| No. 3 | 55.3 ± 1.4 | 428.4 ± 27.9 | <0.001 |
| No. 4 | 1190.3 ± 92.5 | 910.9 ± 181.6 | 0.034 |
| No. 5 | 89.9 ± 32.0 | 73.9 ± 11.7 | 0.566 |

Each study batch was analyzed in at least three independent experiments. Data are presented as mean ± SD. Statistical analysis was assessed by Student's t test.
